# Ultrasonic Measurement of Dynamic Muscle Behavior for Poststroke Hemiparetic Gait

**DOI:** 10.1155/2017/8208764

**Published:** 2017-01-23

**Authors:** Xin Chen, Xudong Zhang, Wenxiu Shi, Jun Wang, Yun Xiang, Yongjin Zhou, Wan-Zhang Yang

**Affiliations:** ^1^School of Biomedical Engineering, Shenzhen University, Shenzhen, China; ^2^National-Regional Key Technology Engineering Laboratory for Medical Ultrasound, Shenzhen, China; ^3^Guangdong Key Laboratory for Biomedical Measurements and Ultrasound Imaging, Shenzhen, China; ^4^Shenzhen Sixth People's Hospital, Shenzhen, China; ^5^Shenzhen Hospital of Southern Medical University, Shenzhen, China

## Abstract

Quantitative evaluation of the hemiparesis status for a poststroke patient is still challenging. This study aims to measure and investigate the dynamic muscle behavior in poststroke hemiparetic gait using ultrasonography. Twelve hemiparetic patients walked on a treadmill, and EMG, joint angle, and ultrasonography were simultaneously recorded for the gastrocnemius medialis muscle. Pennation angle was automatically extracted from ultrasonography using a tracking algorithm reported previously. The characteristics of EMG, joint angle, and pennation angle in gait cycle were calculated for both (affected and unaffected) sides of lower limbs. The results suggest that pennation angle could work as an important morphological index to continuous muscle contraction. The change pattern of pennation angle between the affected and unaffected sides is different from that of EMG. These findings indicate that morphological parameter extracted from ultrasonography can provide different information from that provided by EMG for hemiparetic gait.

## 1. Introduction

Hemiparesis refers to the sensorimotor impairments on only one side of the body commonly observed in poststroke patients [1]. Gait recovery is a major objective in the rehabilitation of hemiparesis patients. Quantitative gait analysis is the best way to evaluate the complex gait dysfunctions that are rela ted to inappropriate control of the central nervous system or changes in the mechanical properties of the muscle [2]. Diverse gait analysis techniques, of which the most commonly used include measurement of temporospatial gait parameters [3, 4], electromyography (EMG) [5], kinematics [6, 7], and kinetics [8], have been developed. In addition, some brain imaging techniques, such as functional magnetic resonance imaging (fMRI) [9] and positron emission tomography (PET) [10], have provided an approach to studying the neural circuitry involved in poststroke motor control. Several articles have reviewed how these techniques have been used to quantify the abnormal patterns in hemiparetic gait [2, 11, 12].

The architecture of skeletal muscle, defined as the geometric arrangement of muscle fibers, is a primary determinant of muscle function. Therefore, assessing the morphological changes in muscle provides an alternative measurement of muscle activity [13]. Since ultrasonography (US) offers the unique advantages of being noninvasive, real-time, and easily accessible, it has been widely applied to measure in vivo changes in muscle during static and dynamic contractions [14–16]. US has been showed to reliably measure changes in muscle thickness [17, 18], fiber length [19, 20], pennation angle [21–23], fascicle curvature [24], and cross-sectional area [16]. Several studies investigated the effect of poststroke impairments on muscle architecture as measured by ultrasound during static contraction [25, 26]. The results showed significant architectural changes in the affected side due to weakness in the muscle after stroke. However, all the previous ultrasonography studies measured the static architectural values under different contraction conditions, and the continuous muscle behavior during hemiparetic gait has not previously been clarified. Although some studies have applied US to investigate the dynamic muscle behavior during human running and walking [27, 28], it has seldom been used in a hemiparetic gait study.

In this study, an experimental and data analysis platform was employed to dynamically capture EMG, joint angle, and ultrasonography signals. The gaits of twelve hemiparetic patients were measured and the characteristics of their muscle behaviors were analyzed. The goal of this study is to investigate the dynamic muscle behavior for poststroke hemiparetic gait using ultrasonography.

## 2. Patients and Methods

### 2.1. Patients

Both chronic and subacute stroke patients were included in this study. A total of twelve patients (9 male and 3 female) participated. Their mean age was 54.9 years (SD = 12.4 years), and the mean poststroke time was 142 days (SD = 196.2 days). The characteristics of the patients are shown in Table 1. The study was approved by the local ethics committee, and all participants gave their written informed consent.

### 2.2. Experimental Procedure

All patients walked on a treadmill at a self-selected speed for 60 s. The walking speed is between 1.0 km/h and 1.5 km/h. In some cases, in order to ensure security, the patients were allowed to hold onto a rail to maintain their balance. For each patient, the experimental procedure was performed twice for test on each of the lower limbs (the affected and unaffected sides). The subjects rested for several minutes after each test.

### 2.3. Data Acquisition and Synchronization

**Figure 1:**
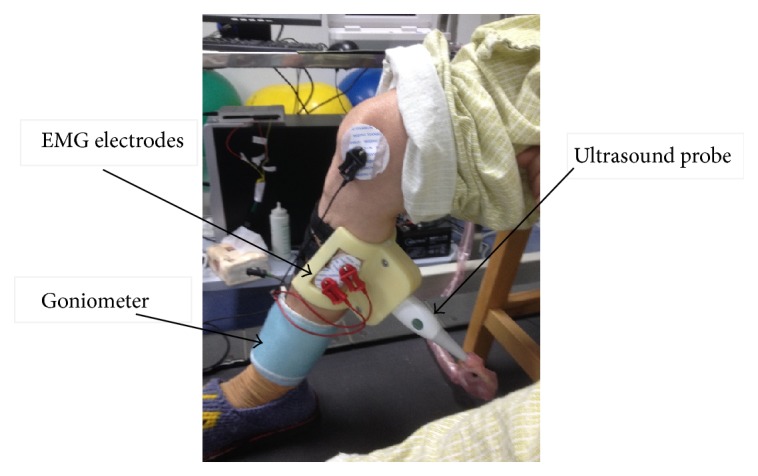
Experimental setup.

The EMG, joint angle, and US were simultaneously recorded from the gastrocnemius medialis (GM) muscle during walking. A real-time B-mode ultrasonic scanner (Sonostar Technologies Co., Guangzhou, Guangdong, China) with an electronic linear array probe was used to obtain longitudinal ultrasound images of the GM muscle. As shown in Figure 1, the ultrasound probe was secured steadfastly by a custom-designed adjustable bracket on the mid-belly of GM muscle. The long axis of the probe was carefully arranged aligning with the middle line of the GM muscle. Ultrasound gel was applied to secure acoustic coupling between the probe and the skin. The B-mode images outputted from the scanner were digitized by a video capture card (Weishi Digital Image Technology Co., Xian, Shanxi, China) with a frame rate of 25 Hz. Surface bipolar Ag-AgCl EMG electrodes (Tianrun Sunshine Medical Supplies Co., Beijing, China) with a diameter of 2 cm were used to capture the EMG signal. Two electrodes were placed on one side of the probe. The line between the centers of the electrodes was parallel to the long axis of the probe. A reference EMG electrode was placed near the kneecap. The joint angle of the ankle was measured by an electronic goniometer (model XM110; Penny & Giles Biometrics, Ltd.; Gwent, United Kingdom) attached in the sagittal plane between the anterior tibia and the dorsum of foot. The surface EMG signal was amplified by an amplifier with a gain of 2,000, filtered by a 10–400 Hz band-pass analog filter (EMG100C, BIOPAC Systems Inc., Goleta, CA, USA). The angle signal was amplified by an amplifier with a gain of 2,000, filtered by a 5–100 Hz band-pass analog filter (DA100C, BIOPAC Systems Inc., Goleta, CA, USA). Both signals were digitized by a 12-bit data acquisition card (DAQ USB-6216, National Instruments Corporation, Austin, TX, USA) with a sampling rate of 1 KHz. The ultrasound images, EMG, and joint angle signals were simultaneously collected and stored for offline analysis using custom-developed software under the LabVIEW environment.

### 2.4. Image Processing

The pennation angle of the GM muscle was extracted from the ultrasound image sequence using an automatic tracking algorithm. Specifically, we enhanced the ultrasound image using Gabor filtering and then used revoting Hough transform (RVHT) to obtain the orientations of the major fascicles and took their mean as the final pennation angle. The details of the algorithm were described elsewhere [21, 23].

### 2.5. EMG and Angle Signals Processing

The EMG signal was segmented into 256 ms epochs. The center of each epoch was aligned in time with the corresponding ultrasound image according to the timestamp, so that the epochs were synchronized with the image sequence in the time domain. The root mean square (RMS) values of the EMG were calculated for each epoch. The angle signal was synchronized with the ultrasound images in the same way.

### 2.6. Statistical Analysis

For each patient, the minimum and maximum values of the joint angle and pennation angle were extracted for each trial. The group means of joint angle and pennation angle were calculated for the trials of both the affected and the unaffected sides. A Kolmogorov-Smirnov (K-S) test was performed to compare the joint angle, EMG, and pennation angle between the affected side and unaffected side. To demonstrate the spatial patterning of the muscle activity, a 2-dimensional histogram based on the distributions of the joint angle and pennation angle was calculated.

For each patient, the EMG, joint angle, and pennation angle signals were manually checked by an experienced operator. The joint angle signal was used as a reference to segment the gait cycles. Eight gait cycles, in which all the three signals have no breaks, were manually selected. For each signal, the time curves in the eight gait cycles were averaged to obtain the mean time curve for each patient. Then the interindividual mean curve among the twelve patients was computed for each signal.

## 3. Results

**Figure 2:**
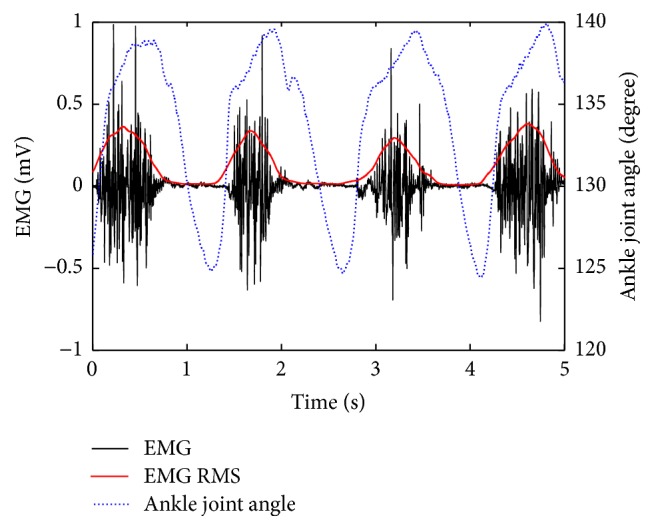
Time curves of the EMG and joint angle during a representative trial on the affected side. The RMS_EMG_ curve is overlapped onto the raw EMG curve.

**Figure 3:**
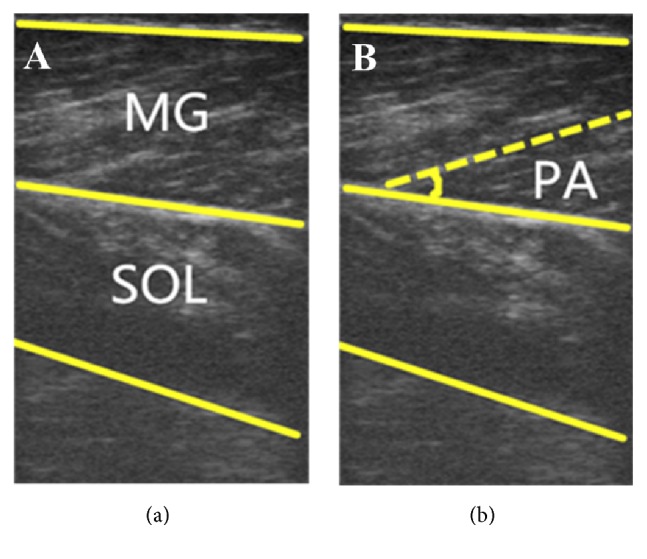
Typical ultrasound images of the medial gastrocnemius muscle during walking.

**Figure 4:**
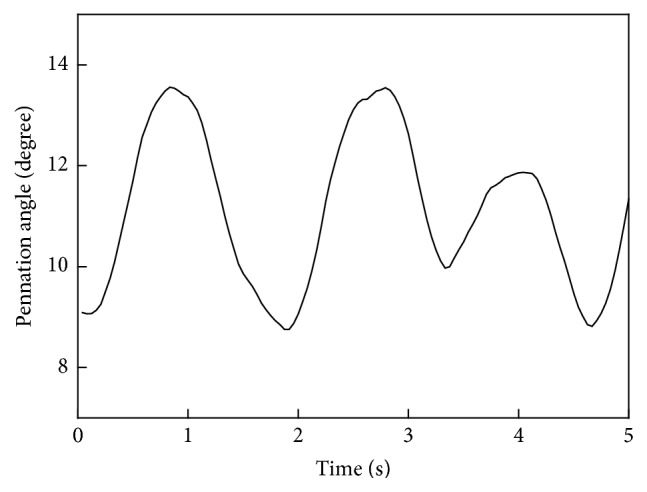
Dimensional change in the pennation angle in a typical trial on the affected side.

**Figure 5:**
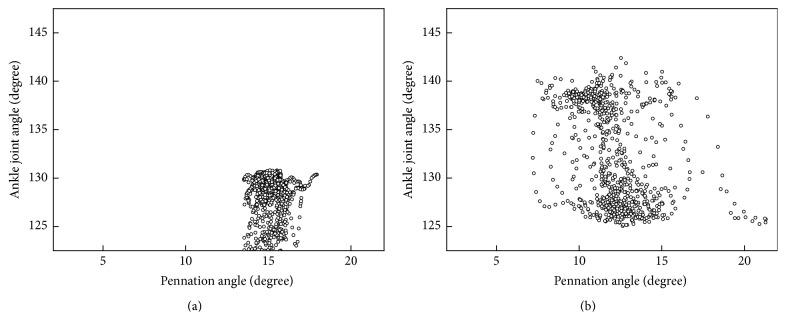
Correlations between the pennation angle and joint angle for (a) the unaffected side and (b) the affected side in a typical trial.

**Figure 6:**
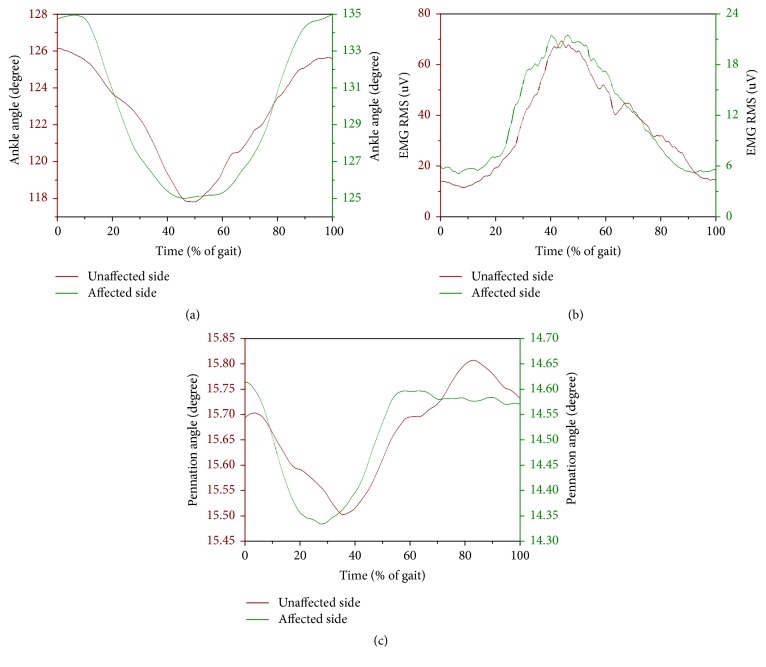
The interindividual mean curve of gait cycle among the twelve patients for (a) joint angle, (b) EMG RMS, and (c) pennation angle for both affected and unaffected sides. For visual clarity, the standard deviation (SD) is not displayed in this figure.

**Figure 7:**
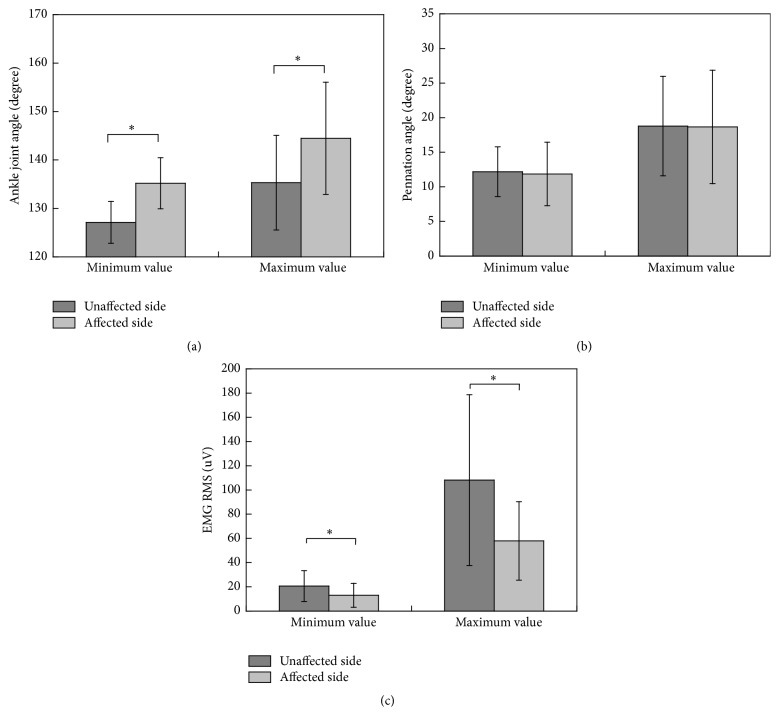
Minimum and maximum joint angle and pennation angle values for both sides during walking. An asterisk indicates that the two groups are significantly different.

Figure 2 shows the EMG and angle signals from one representative trial on the affected side. The RMS_EMG_ curve is also overlapped in the figure. One set of typical US images from the same trial is shown in Figure 3. The corresponding time course of the pennation angle, which was automatically extracted from the ultrasound image sequence, is shown in Figure 4.  Figure 5 shows the scatter plots of the joint angle versus the pennation angle for a typical trial. The interindividual mean curves of gait cycle for the three signals for both affected and unaffected sides are displayed in Figure 6. For visual clarity, the standard deviation (SD) is not displayed in this figure. Instead, the average SD over each curve was calculated. For the unaffected side, the mean SD values are 5.2 degrees for joint angle, 34.4 *μ*V for EMG, and 4.0 degrees for pennation angle. For the affected side, the mean SD values are 7.7 degrees for joint angle, 6.2 *μ*V for EMG, and 1.8 degrees for pennation angle. The comparisons of the joint angle, pennation angle, and EMG between the affected side and the unaffected side are shown in Figure 7. For the comparisons for the minimum values, the *p* values are 0.01, 0.04, and 0.63 for joint angle, EMG, and pennation angle, respectively. For the comparisons for the maximum values, the *p* values are 0.01, 0.03, and 0.62 for joint angle, EMG, and pennation angle, respectively.

## 4. Discussion

In this study, we simultaneously collected US, EMG, and joint angle signals from the GM muscle during hemiparetic gait. The purpose was to examine the contraction patterns of the lower extremity muscle activity and to compare these between the affected and unaffected sides. To the best of our knowledge, this is the first attempt to investigate continuous muscle behavior during hemiparetic gait using ultrasonography.

The conventional techniques for gait analysis are measurement of temporospatial parameters, EMG, kinematics, and kinetics. Of these techniques, kinetic variables refer to moments resulting from forces generated at the joints. This information is particularly helpful in understanding and interpreting the characteristics of stroke gait because kinetic variables are the results of the kinematic and temporospatial outcomes of walking [11]. However, kinetic variables cannot be measured directly but rather are computed by inverse dynamics. In contrast, muscle functions, such as the amplitude of force production, are closely related to architectural characteristics [13]. Therefore, dynamic muscle architectural changes assessed by ultrasonography are more direct measurements than kinetic variables and have the potential to provide more information about stroke gait. Some previous studies have demonstrated that ultrasonography is helpful in measuring the effect of poststroke impairments [25, 26]. However, all the previous ultrasonography studies measured static architectural values at discrete times during contraction. Therefore, continuous muscle behavior during hemiparetic gait has remained unclear. In this study, we developed an image tracking algorithm to automatically extract continuous changes in the pennation angle of the GM muscle, making it possible to identify muscle morphology at a higher temporal resolution.

As shown in Figure 5, the dynamic ranges of the joint angle and the pennation angle were more diffuse for the affected side than for the unaffected side. However, as shown in Figure 7 for the composite mean level, the maximum and minimum values of the pennation angle do not differ between the two sides, but those of the joint angle do. This finding was different from the previous study [29] which reported decreased pennation angle of medial gastrocnemius muscle at separate ankle joint angles. One possible reason for the difference is that our study and that study were conducted under different conditions (dynamic gait and controlled manner, respectively). Although a reasonable explanation for the relationship between the pennation angle and joint angle may involve additional neuromuscular investigations and is beyond the scope of this report which is presented from more of an engineering perspective, it is encouraging to see that the proposed method has made it possible to reveal such findings. Another advantage of the proposed method is that US signal can reveal more information about the hemiparetic pattern than the conventional EMG signal. As shown in Figure 6, the EMG curves for affected and unaffected sides have similar waveforms with significant amplitude difference. The pennation angle curves for affected and unaffected sides have relatively small amplitude difference. Instead, the two curves have significantly different timing. These findings indicate that morphological parameter extracted from ultrasonography can provide different information than that provided by EMG.

The experiment setup in this study has several shortcomings. First, though we tried to keep the probe in the same imaging plane via visual feedback from one sampling time to the next, the imaged section of the 3D muscle structure was not completely consistent. Consequently, the pennation angle estimations could have included inaccurate values. In other words, it is impossible for longitudinal imaging of a muscle to adequately reflect the 3D motion of the muscle. Better imaging facilities and setup, as well as more comprehensive modeling, can help to disclose the pennation angle dynamics in ultrasound images in future studies. This problem is actually common to all current 2D US methods. Second, we did not use a fixed walking speed for all the patients. It is well known that speed has strong influences in the gait pattern, in respect to both kinematics and kinetics. However, each patient has his own adapted speed and he may not be used to another speed without training. Therefore patients walked at a self-selected speed in this study. At last, the ankle angle is conventionally defined as positive values for dorsiflexion and negative values for plantar flexion. However, this angle is difficult to measure because it requires attachment of goniometer sensor to bottom of foot. In this study, we measured the angle between the anterior tibia and the dorsum of foot as alternative for ankle angle. The two angles are directly related and both of them can be used as reference for gait cycle.

## 5. Conclusion

In this study, the gaits of twelve hemiparetic patients were measured and the characteristics of their muscle behaviors were analyzed. The results suggested that morphological parameter extracted from ultrasonography can provide different information from that provided by EMG. Thus, ultrasonography can be a useful additional tool for the assessment of hemiparesis patients in clinical applications.

## Figures and Tables

**Table 1:** Patient characteristics.

Subject	Age (year)	Gender	Time poststroke (day)	Affected side	Brunnstrom motor stage
1	45	Male	50	Left	3
2	60	Male	130	Right	4
3	45	Male	25	Left	4
4	46	Male	211	Right	4
5	44	Female	197	Left	3
6	62	Female	64	Left	4
7	47	Male	119	Left	3
8	69	Male	731	Right	5
9	77	Male	53	Right	4
10	55	Male	76	Right	4
11	70	Female	30	Right	5
12	39	Male	18	Right	3
